# HN1 Functions in Protein Synthesis Regulation via mTOR‐RPS6 Axis and Maintains Nucleolar Integrity

**DOI:** 10.1111/cpr.13805

**Published:** 2025-01-13

**Authors:** Gülseren Özduman, Aadil Javed, Azime Akçaöz Alasar, Bünyamin Akgül, Kemal Sami Korkmaz

**Affiliations:** ^1^ Faculty of Engineering, Department of Bioengineering, Cancer Biology Laboratory Ege University Bornova Izmir Turkey; ^2^ University of Michigan, Department of Neurosurgery, Michigan Medicine University of Michigan Ann Arbor Michigan USA; ^3^ Faculty of Science, Department of Molecular Biology and Genetics Izmir Institute of Technology Urla Izmir Turkey

**Keywords:** HN1, mTOR, nucleolin, nucleolus integrity, ribosome biogenesis, RPS6

## Abstract

Haematological and Neurological Expressed 1 (HN1) is an oncogene for various cancers and previously has been linked with centrosome clustering and cell cycle pathways. Moreover, HN1 has recently been reported to activate mTOR signalling, which is the regulator of ribosome biogenesis and maintenance. We explored the role of HN1 in mTOR signalling through various gain‐ and loss‐of‐function experiments using biochemical approaches in different cell lines. We demonstrated for the first time that HN1 is required for nucleolar organiser region (NOR) integrity and function. Immunoprecipitation‐based association and colocalization studies demonstrated that HN1 is an important component of the mTOR‐RPS6 axis, and its depletion results with reduced mRNA translation in mammalian cancer cell lines. This study also demonstrated that the depletion of HN1 leads to the irregular distribution of nucleolar structures, potentially leading to cell cycle deregulation as reported previously. Accordingly, components of the translation machinery aggregate with a distinct speckled pattern, lose their essential interactions and ultimately impair mRNA translation efficiency when the HN1 is depleted. These results suggest that HN1 is an essential component of the nucleolus, required for ribosome biogenesis as well as global mRNA translation.

## Graphical abstract

HN1 is required for nucleolar homeostasis which is the site of ribosome biogenesis and protein synthesis when its mRNA is knocked down by shRNA, it depletes in cytoplasm and the protein synthesis is downregulated in comparison to controls. Thus, the proliferating cells requiring vast number of nucleosomes, are arrested at S onset.

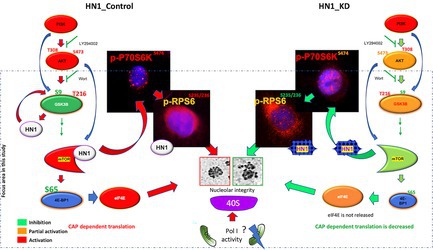

## 1 Introduction

Haematological and Neurological Expressed 1 (HN1) is a multifunctional protein with emerging significance in cellular homeostasis and oncogenesis [1]. Initially identified in murine haematological and neurological tissues [2], HN1 has since been characterised as an oncogene implicated in various cancers [3, 4, 5]. It is also known as Jupiter homologue 1 (JPT1), based on homology with a similar protein discovered in *Drosophila melanogaster* [6, 7]. Its functions are primarily associated with key cellular processes, including cell cycle regulation, centrosome clustering and microtubule dynamics [1]. HN1 is phosphorylated by GSK3β and Cdk1 and partially localises to the centrosome and perinuclear regions in cancer cell lines [8, 9]. Its ubiquitous expression is kept throughout various cell types, implying that HN1 functions in eukaryotic cell survival since metazoan evolution as it is conserved across species [7]. It is also implicated in growth, proliferation, and wound healing, along with the promotion of invasion when overexpressed, whereby its tumour‐promoting function was also reported in hepatocellular cancer, anaplastic thyroid cancer, ovarian cancer and melanomas, consistent with higher expression in various tumour types [3, 5, 10, 11].

Stress stimuli in eukaryotic cells often induce protein granulation near the nucleolar region, regulated by pathways involving the proteasome and protein synthesis machinery [12]. While these processes are essential for controlling stress and maintaining cell survival, the mechanisms underlying stress‐induced granulation and its connection to cellular responses remain poorly understood [13]. Major pathways required for energy sources for protein synthesis involve GSK3β and mTOR regulators [14]. The mTORC1 and mTORC2 complexes converge the signals by sensing the divergent cellular pathways such as nutrient, or amino acid depletion. A recent study reported that HN1 is associated with mTOR in ovarian cancer cell lines, and its depletion via shRNA almost completely suppressed tumour formation in mouse xenograft models [15]. The influence of GSK3β and mTOR signalling on protein synthesis machinery and the role of HN1 in these signalling pathways warrant an investigation of HN1 in the context of protein synthesis mechanisms. Here, our study focused on the putative function of HN1 in the regulation of nucleoli (sites of ribosome biogenesis), whereby the process involves various regulatory and structural components in the nucleolus. We explored the phenotypes of HN1‐altered cells for the integrity of the nucleolar organiser region (NOR) and mTOR‐P70S6K1‐RPS6 axis to determine how HN1 is involved in pathways regulating protein synthesis and turnover.

## 2 Materials and Methods

### 2.1 Cell Culture Propagation and Synchronisation

The MDA‐MB231 and PC3, as well as androgen‐responsive prostate cell line LNCaP and normal mammary gland epithelial MCF10A cells, were obtained from the American Type Culture Collection (ATCC; Manassas, VA). The cell lines were propagated as recommended in either RPMI 1640 or DMEM/F12 supplemented with 10% and 5% fetal bovine serum (FBS) respectively, along with L‐glutamine (2 mM), penicillin (100 U/mL) and streptomycin (100 μg/mL) at 37°C in a humidified atmosphere with 5% CO_2_. Cells were checked routinely for mycoplasma by either PCR or DAPI staining.

### 2.2 Transfections and Ectopic Expression of HN1

The HN1 cDNA sequence was fully amplified using custom‐designed forward and reverse primers (10 pmol each) with the aid of LightCycler Probe Design Software 2 (Roche, Germany). The amplified PCR product was then inserted into the pcDNA4‐HM‐TOPO vector (Invitrogen, UK) according to the manufacturer's protocol, resulting in the creation of the pcDNA4‐HM‐HN1 construct. The size of the constructs was confirmed through PCR amplification. These constructs will be referred to as either the HM vector or HM‐HN1. Subsequent transfections were carried out using the Fugene HD transfection reagent (Roche, Germany), following the manufacturer's recommended protocols. In brief, cells were plated in 60‐mm dishes 24 h before transfection. The following day, a transfection mixture was prepared by diluting 3 μL of the transfection reagent in 100 μL of pre‐warmed DMEM, incubating for 5 min, and then adding 1 μg of plasmid DNA also diluted in 100 μL of pre‐warmed DMEM. After a 15‐min incubation at room temperature (RT), the mixture was gently added to the cells in a dropwise manner.

### 2.3 Establishment of Stable HN1 Knockdown (KD) and HN1 Overexpressing (OE) Cell Lines

The HN1 gene was isolated from the HM‐HN1 construct using Sma1 and EcoRV enzymes and then cloned into the pCW57.1 plasmid, which had been previously digested with Nhe1 and Sal1 enzymes and kindly provided by Dr. Şerif Sentürk. The pCW57.1‐HM‐HN1 plasmid was then co‐transfected alongside packaging plasmids (pMD2G, pRSV‐Rev and pMDLg/pRRE) into HEK293T cells at a 1:3 ratio (μg DNA:μL PEI) in DMEM/F12 medium supplemented with 5% FBS, following standard cell culture protocols. To boost transfection efficiency, cells were pretreated with chloroquine (25 μM) 5 h before transfection. The viral supernatant was then harvested at 48‐ and 72‐h post‐transfection, subjected to centrifugation at 16,000 g for 18 h, and finally resuspended in 100 μL of PBS solution. Next, cells appropriate for transduction were transduced with pCW57.1‐HM‐HN1 virus particles in the presence of polybrene (10 μg/mL) and then selected using puromycin (1–2 μg/mL, depending on the data from the kill curve for each cell line) treatment for 6 days. Following this, both empty vector and pCW57.1‐HM‐HN1 transduced PC3 cells were cultured, and the induction of HN1 by the doxycycline (1 μg/mL) treatment was confirmed through western blot analysis at various time points in pCW57.1‐HM‐HN1 cells. For cell lines stably depleted for HN1, shHN1 in lentiviral backbone plasmid bought from Santacruz Biotechnology was packaged in lentiviruses using the method explained above. The cells were transduced with shHN1 particles also as described for HN1 stably expressing cells.

### 2.4 Treatments With Specific Inhibitors

Cells were treated with Proteosome inhibitors MG132 (10 μM), and Bortezomib (100 nM), along with PI3K inhibitor LY294002 (25 μM), Akt inhibitor wortmannin (100 nM), RNA polymerase inhibitor Act D (200 ng/mL) and GSK3β beta inhibitor SB216763 (10 μM) for appropriate periods. The cell cycle inhibitors were also used to induce arrest at either S or G2/M using 20 μM Thymidine (double block as appropriate) and/or 165 nM nocodazole (at 16–24 h), respectively for further studies.

### 2.5 RNA Extraction and PCR

Cells (1 × 10^5^) were plated from the same batch of experiments of polysome analysis for shcontrol and shHN1, and cells were washed once with RLT buffer and transferred into 350 μL RLT buffer with B‐mercaptoethanol until isolation. MinElute column purification kit was used for RNA isolation according to the manufacturer's recommendations. (Invitrogen, CA, USA). One microgram of purified RNA was used for cDNA synthesis together with random oligos (1 pmol) and Superscript III RTase kit, according to the manufacturer's recommendations (Invitrogen, CA, USA). For the PCR reaction, 2 μL of the cDNA (100 ng) template, forward and reverse primers (5 pmol each) (Table 1) were mixed with 2XSYBR Green mix (Invitrogen, CA, USA), and reactions and CP calculations were performed using Roche LightCycler 480 (Roche, Germany) and the software respectively.

**TABLE 1:** The list of primers used in this study.

Primers	18S rRNA	28S rRNA
F1	5′‐CTGAGAAACGGCTACCACAT	5′‐GACCCGAAAGATGGTGAACT
R1	5′‐TGCCCTCCAATGGATCCTC	5′‐GCCGGGCTTCTTACCCATT
F2	5′‐TGGTGGAGCGATTTGTCTG	5′‐CCTAGTGGGCCACTTTTGG
R2	5′‐TGAGCCAGTCAGTGTAGCG	5′‐GCGACGCTTTCCAAGGCA

### 2.6 Immunoblotting

Cells were lysed using ice‐cold RIPA buffer (containing 1% Nonidet P‐40, 50 mM Tris–HCl at pH 7.4, 0.25% Na‐deoxycholate and 150 mM NaCl) supplemented with 1 mM NaF, 1 mM EDTA and 1 mM Na_3_VO_4_, and complete protease and phosphatase inhibitors and cocktails (obtained from Roche, Germany), unless specified otherwise. Proteins were resolved by electrophoresis on 10%–15% SDS‐polyacrylamide gels and then transferred to PVDF membranes (Amersham, UK) using a wet transfer blotter. The membranes were subsequently blocked with TBS‐T (Tris‐buffered saline containing 0.1% Tween‐20) supplemented with 5% skim milk (w/v) to prevent non‐specific binding. Antibody incubations were carried out in TBS‐T containing 0.5% dry milk for either 1 h at RT or overnight at 4°C. Subsequently, the membranes were developed using the ECL Plus reagent (Amersham, UK) for 5 min, and the resulting chemiluminescent signals were captured on Kodak x‐ray film in a dark room. Antibodies used at concentrations of 0.2–1 μg/mL, where appropriate in this study are given in Table 2.

**TABLE 2:** The list of antibodies used in the study.

Antibody	Technique	Manufacturer and catalogue	Dilution
Anti‐HN1	IB, IF, IP	Invitrogen—PA5109824	1/750, 1/300, 2 μg
Anti‐Cdk2	IB	Santa Cruz—sc‐6248	1/500
Anti‐β‐Actin	IB	Sigma—A3854	1/500.000
Anti‐ p62	IB	Proteintech—18,420‐1‐AP	1/10000
Anti‐β‐catenin	IB	Santa Cruz—sc59737	1/500
Anti‐Cdk1	IB	Abcam—ab131450	1/1000
Anti‐p‐4EBP1^(S65)^	IB, IF	Santa Cruz—sc‐293,124	1/500, 1/200
Anti‐4EBP1/2/3	IB	Santa Cruz—sc‐271,947	1/500
Anti‐4EBP1	IB	Santa Cruz—sc‐9977	1/500
Anti‐p‐GSK3β^(S9)^	IB	Santa Cruz—sc‐11,757	1/500
Anti‐p‐GSK3β (Tyr 216)	IB	Santa Cruz—sc‐135,653	1/500
Anti‐p‐p70S6K1^(S434)^	IB, IF	Santa Cruz—sc‐8416	1/500, 1/200
Anti‐p‐RPS6^(S235/236)^	IB, IF	Santa Cruz—sc‐293,144	1/500, 1/200
Anti‐RPS6	IB, IF	Santa Cruz—sc‐74,459	1/500, 1/200
Anti‐UBF	IB, IF	Santa Cruz—sc‐13,125	1/500, 1/200
Anti‐mTOR	IB	Santa Cruz—sc‐517,464	1/500
Anti‐nucleolin	IB, IF	Santa Cruz—sc‐8031	1/500, 1/200
Anti‐BrdU	IF, FC	Santa Cruz—sc‐32,323	1/200, 1/200
Anti‐GAPDH	IB	Ambion—AM4300	1/240.000
Anti‐lamin A/C	IB	Santa Cruz—sc‐376,248	1/500
Anti‐rabbit	IB	Jackson Immuno Research—111‐035‐003	1/25.000
Anti‐mouse	IB	Jackson Immuno Research—515‐035‐003	1/25.000
Alexa488 (anti‐mouse IgG)	IF	Thermo A‐21202	1/600
Alexa594 (anti‐mouse IgG)	IF	Thermo A‐21203	1/600
Alexa488 (anti‐rabbit IgG)	IF	Thermo A‐11008	1/600
Alexa594 (anti‐rabbit IgG)	IF	Jackson Immuno Research 711‐585‐152	1/600

### 2.7 Subcellular Fractionation

Briefly, the cells were collected either by scraping or adding trypsin, pelleted at 300 g for 5 min at RT after treatments. They were washed with PBS, resuspended in Buffer A (1 mM EGTA, 1 mM EDTA and 50 mM HEPES pH: 7.4, and 10 mM KCl) and placed on a rotator for 30 min at 4°C. Cytoplasmic fraction was collected from the supernatant after centrifugation at 4000 g for 5 min at 4°C. The pellet was washed in Buffer A four times to remove residual cytoplasmic proteins. After washing, pellets were resuspended in Buffer B (0.5% Triton‐X‐100, 1 mM EGTA, 1 mM EDTA, 1 M HEPES pH: 7.4 and 400 mM KCl) and shaken on a rotator for 30 min at 4°C. Then, the mixtures were centrifuged at 14000 g for 30 min at 4°C. The supernatant was collected as the nuclear fraction. For isolating chromatin fraction separately, the nuclear lysate was centrifuged, resuspended in RIPA (1% Nonidet P‐40, 50 mM Tris–HCl [pH 7.4], 0.25% Na‐deoxycholate and 150 mM NaCl) and sonicated. The protein concentration from nuclear, cytoplasmic and chromatin fractions was measured as described before [8]. Anti‐GAPDH and anti‐lamin antibodies were used as loading controls for cytoplasmic and nuclear lysates, respectively.

### 2.8 Polysome Analysis

Polysome analysis was performed essentially as previously described [16]. Cells were incubated with 50 μg/mL cycloheximide (CHX) for 10 min in a humidified atmosphere of 5% CO_2_ at 37°C prior to washing with 1X RNase‐ and DNase‐free chilled PBS. Cells were trypsinized and centrifuged at 1000 rpm for 5 min. 4 × 10^6^ cells were then resuspended in lysis buffer (100 mM NaCl, 10 mM MgCl2, 1% Triton X‐100, 1% sodium deoxycolate (NaDoC), 30 mM Tris‐Cl, pH 7.5, 100 μg/mL CHX and 30 U/mL RNasin Ribonuclease inhibitor [Promega]) and passed through a syringe with a 26 G needle at least 15 times. The cell homogenates are incubated on ice for 8 min and then centrifuged at 12,000 × g at 4°C for 15 min. The resulting supernatants were layered over 5%–70% (w/v) sucrose gradients and centrifuged at 27,000 rpm for 2.5 h at 4°C using a Beckman SW28 rotor (Beckman Coulter). Fractions were collected continuously from the top of the gradients while monitoring the absorbance at 254 nm using the ISCO Teledyne density gradient fractionation system.

### 2.9 Immunoprecipitations

Protein lysates were obtained for immunoprecipitation as mentioned for immunoblotting. 0.5 mg protein lysate was pre‐cleared in the IP‐matrix (40 μL) (Santa Cruz Biotechnology Inc.) or Dynabeads protein G (10003D, Thermo Fischer Scientific, USA) according to the manufacturer's recommendations. Briefly, lysates were divided into two parts and used for the reaction with either a specific antibody against a protein of interest or a non‐specific immunoglobulin G for 4 h at 4°C. The IP matrix (40 μL) or magnetic beads (20 μL) were then used to form complexes with the antibody in the reaction of the antibody and the lysate overnight at 4°C before washing with a RIPA‐modified buffer. The complexes were washed thoroughly in a RIPA‐modified buffer four times before denaturation of the samples in a Laemmli buffer (25 μL) for 5 min at 95°C. The solution was then run on SDS gel for protein separation and immunoblotted for target antibodies as specified previously.

### 2.10 Flow Cytometry

To determine the cell cycle distribution of the cells with various inhibitor treatments or BrdU, the cells were harvested by trypsinization, fixed in 70% ethanol in PBS and stored at −20°C for at least 24 h. Then, the indirect labeling was performed with primary and Alexafluor‐conjugated secondary antibodies in stable HN1 KD and/or OE cell lines. Briefly, fixed cells were washed with PBS and incubated with 0.2% Triton X‐100 in PBS for 5 min at RT on a shaker. Cells were washed with PBS and incubated with 1% bovine serum albumin (BSA) for 5 min. After incubation with primary antibody for 1 h at 37°C, two rounds of PBS washes were performed. Then the cells were incubated with a secondary antibody in 1% BSA for 30 min at 37°C. After two rounds of washes, the cells were pelleted and resuspended in 20 μg/mL RNAse A solution in PBS and incubated at 37°C for 30 min. Finally, cells were pelleted again and resuspended in 1 μg/mL propidium iodide (PI) in PBS. The cells were analysed with a C6 BD Accuri flow cytometer (Becton Dickinson, USA) and cell cycle distribution was calculated using the FlowJo v10 software.

### 2.11 Immunofluorescence (IF) Labeling and Microscopy

Treated cells grown on coverslips were fixed in either cold methanol (100%) at −20°C for 30 min or paraformaldehyde (4% in PBS) at RT for 30 min, were washed with PBS and were permeabilized in 0.2% Triton X‐100 in PBS for 5 min at the shaker, before next washing. Cells were blocked by incubation in 1% BSA in PBS for 5 min on a shaker at RT. Cells were then incubated in primary antibody dilutions prepared in 1% BSA in PBS for 1 h in a humidified chamber at 37°C. Cells were washed with PBS four times before secondary antibody incubation for 20 min in the same conditions as primary antibody incubations. The secondary antibodies were either Alexafluor 534 (anti‐mouse), Alexafluor 594 (anti‐mouse) and/or Alexafluor 488 (anti‐rabbit)‐conjugated antibodies (Invitrogen, Carlsbad, USA). After incubation, cells were washed with PBS four times and treated with 70% ethanol for 1 min and 100% ethanol for 1 min. The cover glasses were then air‐dried and mounted on glass slides with 0.5–1 μg/mL DAPI in 30% glycerol in PBS and analysed immediately under a DM4000 LED B fluorescence microscope (Leica, Germany) as described previously [17]. Images were captured with a 5.5 Mpix digital camera and analysed with the Leica imaging software 4.12.

### 2.12 Cell Proliferation Assay

To evaluate cellular proliferation upon SB216763, MG132 and Bortezomib treatments to shHN1 cells, an MTT [3‐(4,5‐dimethylthiazol‐2‐yl)‐2,5‐diphenyltetrazolium bromide] assay was performed. Briefly, 1 × 10^4^ cells were split into 96‐well plates containing growth medium. After 24 h, different concentrations of treatments (2.5–20 μM for SB216763 and MG132; 25–200 nM for Bortezomib) were applied. DMSO was used as a MOCK control. After 44 h of treatments, 0.5 mg/mL MTT was added to the cells and incubated at 37°C for 4 h additionally. Then, the MTT‐containing medium was removed, and 100 μL of DMSO was added to each well to form the aggregates to be solubilised; the absorbance at OD570 nm was read in a microplate reader (Thermo Scientific, USA).

### 2.13 Statistical Analysis

The data graphed in bar or line graphs are taken as the means ± standard error of the mean (SEM) for independent setups for the analyses and are shown in either main figures or Supporting Information figures. For visualisation, Microsoft Excel or GraphPad Prism 9 software packages were used. Where applicable, the differences in variances are recorded according to *p* value (< 0.05 or lower where necessary) based on ANOVA, two‐way ANOVA or multiple unpaired t‐tests to show the statistical significance (*).

## 3 Results

### 3.1 The Effect of Protein Synthesis and Proteasome Inhibitors on HN1 Expression

**FIGURE 1:**
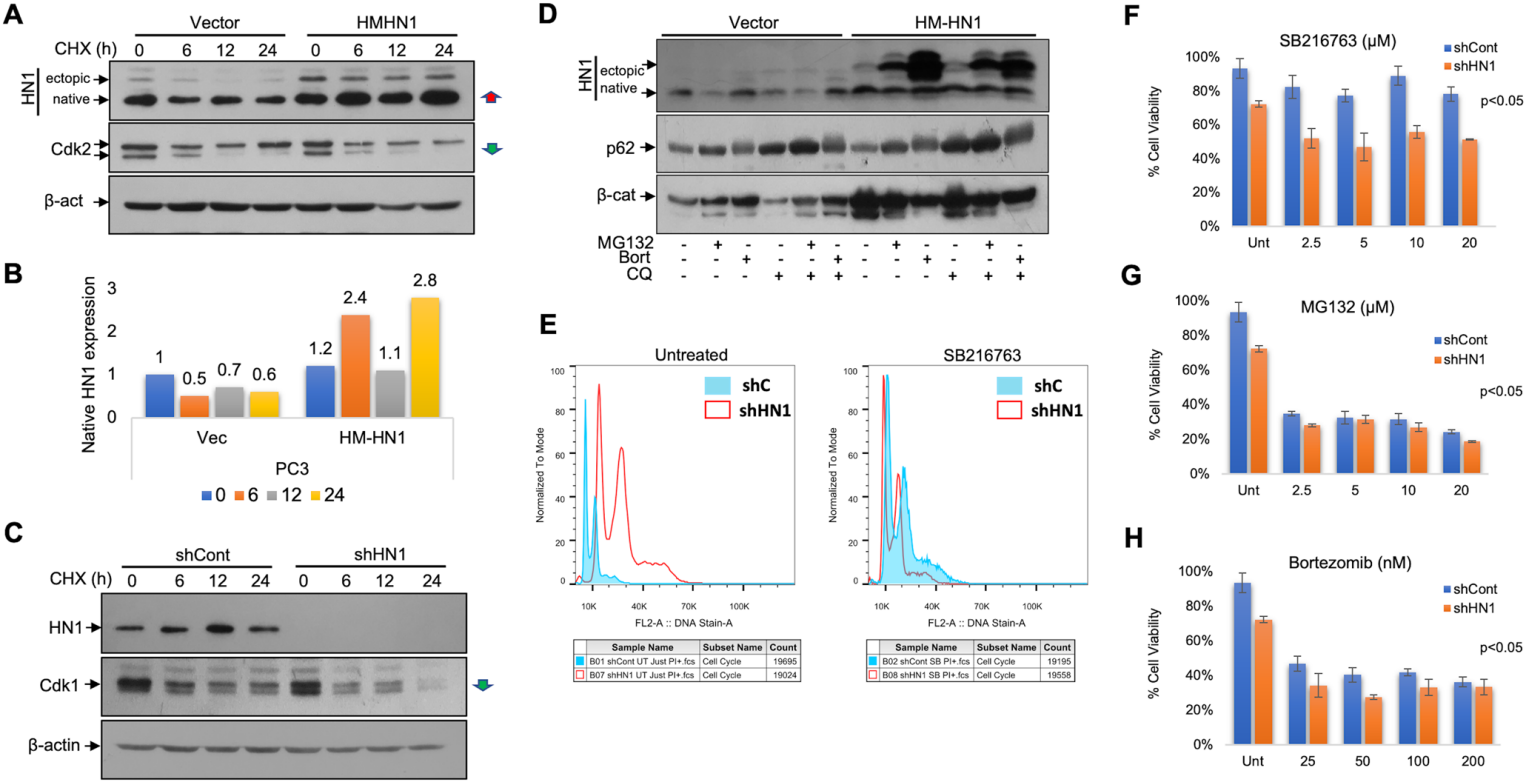
(A) Changes in the half‐life of the proteins were investigated using a Cycloheximide (1 μM) treatment time course (0–24 h) in cells with stabilised HN1 expression (OE) and found that the HN1 OE increased only the native HN1 abundance but did not influence Cdk2 and B‐actin proteins' half‐life significantly. The increases in expressions were noted with a red arrow and the decreases with a green arrow respectively. (B) Native HN1 expression stabilisation was quantitated from Western data and graphed. The ectopic expression of HN1 stabilised native form more than 2‐fold despite CHX treatment. (C) shRNA mediated knockdown (KD) of HN1 expression reduced the half‐life of the proteins tested significantly, where the decreases in expressions were shown in green arrows. (D) Ectopic HN1 expression increased native HN1's stability in PC3 cells. β‐catenin and p62 were used as controls for proteosomal and autophagy‐mediated degradations respectively. (E) GSK3B inhibitor SB216763 was given to shRNA‐depleted HN1 KD PC3 cells in comparison to controls and flow cytometry analysis was performed to evaluate the cell cycle phases. (F) HN1 depletion reduced the cell viability by almost 20% (*p* < 0.05) even in untreated cells. The shRNA‐mediated cell growth inhibition is augmented by GSK3Bi and proteosome inhibitors, (G) MG132, (H) bortezomib treatments. The cell viability significantly decreased further down to 40% with inhibitors, concentration‐dependently (*p* < 0.05).

First, the stability of HN1 in cancer cell lines as well as in normal epithelial cells was investigated using 1 μM CHX with time course treatments (0–24 h). In the cells expressing HN1 (named as OE) ectopically (tet‐on cells induced with doxocycline), the Cdk2 (S phase kinase) expression decreased gradually to 24 h as expected (Figure 1A). However, we observed that the ectopic HN1 expression did not decrease by CHX time course, instead accumulated marginally and interestingly, resulting in the stabilisation of the native HN1 expression more than 2‐fold despite CHX treatment (Figure 1A,B). Increases and decreases in expression were indicated with a red and green arrow, respectively. Consistently, when we performed HN1 KD experiments, we found that HN1 KD shortened the half‐life of Cdk1 significantly in control cells (Figure 1C). HN1 OE and silencing effects for CDK's expressions at 0 h samples were consistent with our previous studies [9]. To further investigate how the HN1 expression is maintained, HN1 stabilisation was examined in the presence of proteasome and autophagy inhibitors with appropriate controls (p62 for autophagy and β‐catenin for proteosome). As expected, native HN1 expression was accumulated only upon Bortezomib treatment, and its ectopic expression (HN1 OE) augmented the native HN1's stability consistently. HN1 overexpressed form (larger in size) was also stabilised upon proteosome inhibitor treatments (Figure 1D). In another experimental setting, proteosome inhibitor MG132 and protein synthesis inhibitor CHX were used together (in a time course of CHX) to block the protein degradation and the synthesis at certain time points. Here, we expected to see a time frame in which native HN1 expression would accumulate and degrade. We repeatedly observed that native HN1 accumulated and degraded and then stabilised in a time frame of 8–16 h in larger‐size protein forms (this experiment was repeated twice independently). Since the rate of HN1 accumulation varied in different cell lines, but most likely the larger bands were phosphorylated forms in both cell lines studied, we expected a biphasic accumulation and depletion of HN1 forms in time, as we observed in our previous studies [8]. One of these bands was GSK3β phosphorylation. Therefore, we suggested that the accumulations might be related to post‐translational regulation of HN1 (Figure S1A). While MG132 partially protected HN1 from proteosomal degradation, and the CHX treatment blocked the protein synthesis, we examined if HN1 levels are protected after its phosphorylation via GSK3β inhibition. To test this hypothesis, we constructed a site‐directed mutant of HN1 at the putative phosphorylation site of HN1, Δ(S87‐92) using pcDNA4‐HM‐wtHN1‐ORF construct. Then the wtHN1 and HN1Δ(S87‐92) were used to ectopically express wtHN1 and its phospho site deletion mutant Δ(S87‐92). We observed that all ectopic_wt and mutant, as well as native expressions of HN1, decreased upon treatment with specific GSK3β inhibitor SB216763. The decrease was not related to HN1 phosphorylation at least at HN1Δ(S87‐92) sites but the GSK3β inhibition resulted in a significant depletion of HN1 expression (Figure S1B). To check whether this might be related to cell cycle delay upon SB216763 treatment or the HN1 expression profile, the populations were examined in shRNA‐depleted HN1 KD cells in comparison to controls using flow cytometry. Here, we found that the significantly changing cell cycle phase ratios in KD cells, in comparison to controls, were suppressed by SB216763 treatments in PC3 cells (Figure S1C). Additionally, the nuclei size increased more significantly in control PC3 cells 2*n*/4*n* from 6/12 to 14/28 K in comparison to SB216763 treatments 2*n*/4*n* from 12/24 to 9/18 K. When another cell line DU145 was examined, the cell size changed 2*n*/4*n* from 40/80 to 64/128 K, and with SB216763 treatments 2*n*/4*n* from 44.6/89 to 56/112 K (histogram plot is not shown). Therefore, we proposed that the nuclear size changes upon HN1 depletion, and it is suppressed by GSK3β inhibitor in PC3 cells (Figure 1E).

As it is previously reported that overall protein abundance in cancer cells is affected as a result of tumorigenesis [18] and consistently HN1 KD cells exhibited a lower growth rate than controls (Figure S2A), we examined and found that the colony formation rate (Figure S2B,C), and the invasion rate were lower than controls as quantitated using real‐time Boyden chambers (Figure S2D,E). Accordingly, we proposed that the overall protein synthesis might be affected by HN1 depletion. The cellular migration rate was also measured using scratch assay and found that it was also slower in KD cells than in controls (Figure S2F,G), suggesting that the cellular survival is suppressed by depleted HN1 expression. To determine whether the HN1 expression is essential for cell survival via protein synthesis, we treated HN1 KD and control cells with SB216763 as well as proteosome inhibitors. We observed that the cellular growth inhibition by HN1 depletion was almost 20% (*p* < 0.05) and is augmented by GSK3βi and proteosome inhibitors. Thus, the cell viability is significantly decreased concentration‐dependently further down to 40% with inhibitors (*p* < 0.05) in addition to HN1 depletion (Figure 1F–H). We proposed that the HN1 expression abundance influences protein turnover, which warrants further studies.

### 3.2 HN1 Contributes to 4EBP1 Phosphorylation

**FIGURE 2:**
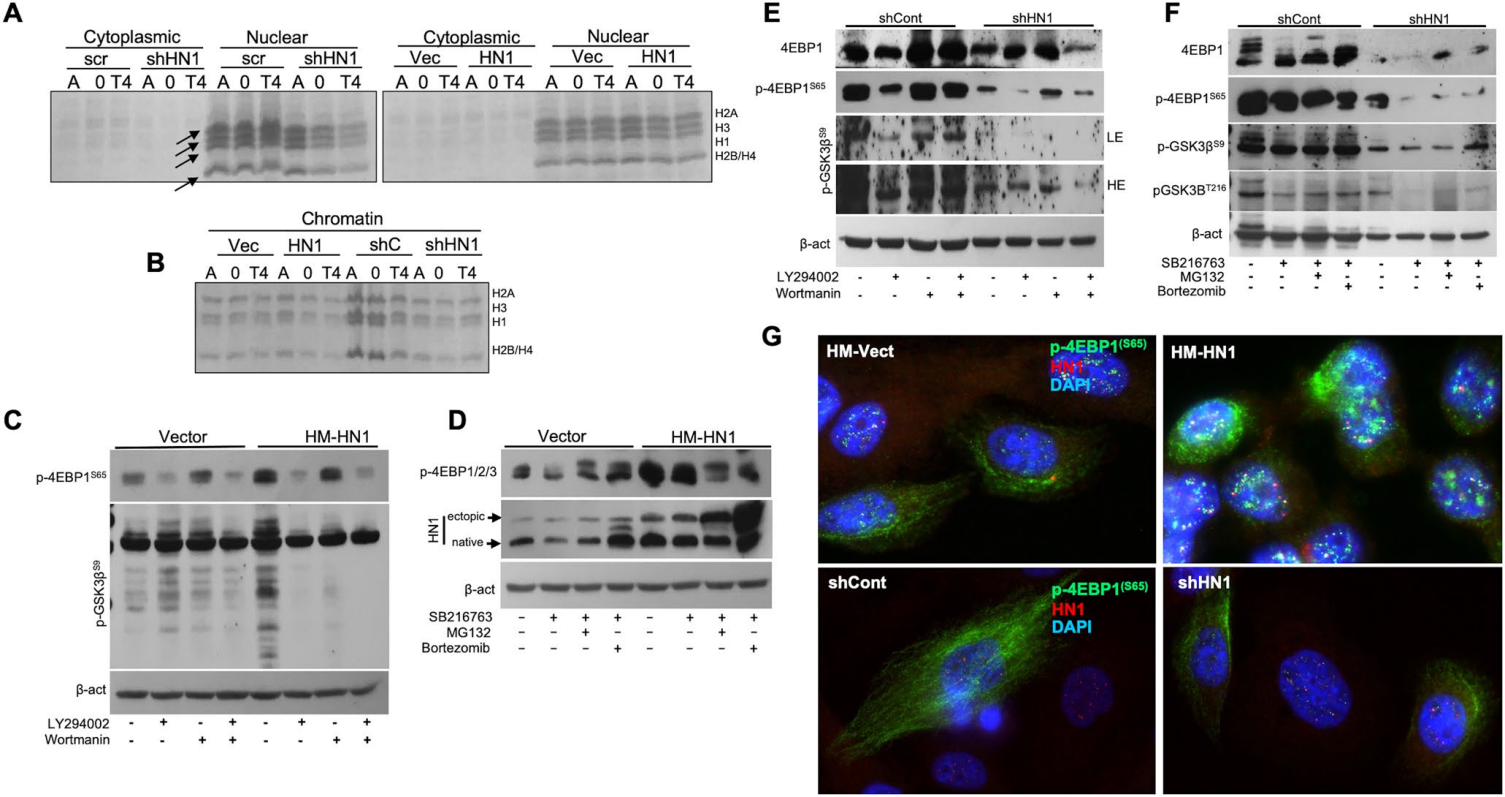
(A) Subcellular fractionation was performed in PC3 cells when HN1 KD or OE. Asynchronous or Thymidine synchronised cells were used to evaluate the timing of alteration when HN1 expression is changed. It is observed that histone protein expressions decreased in HN1 KD cells, but not in OE cells significantly in comparison to controls. (B) The observation was repeated with chromatin‐enriched lysates. Intriguingly shHN1 cells exhibited lower‐sized histones in Coomassie gels in both KD and OE phenotypes. (C) p‐GSK3β^(S9)^ and p‐4EBP1^(S65)^ phosphorylations were studied w/wo Akti (wortmannin) and PI3Ki (LY294002) treatments for 24 h in HN1 OEcells. Clearly HN1 OE stabilised both p‐GSK3β^(S9)^ and p‐4EBP1^(S65)^ phosphorylations. (D) p‐4EBP1/2/3 phosphorylations were also examined w/wo GSK3Bi (SB216763) treatment and observed that HN1 OE increased p‐4EBP1/2/3 phosphorylations and proteosome inhibitors MG132 and bortezomib stabilised the protein levels as well as phosphorylations in HN1 OE PC3 cells in comparison to vector controls. (E) p‐GSK3β^(S9)^ and p‐4EBP1^(S65)^ phosphorylations were studied w/wo Akti (wortmannin) and PI3Ki (LY294002) treatments for 24 h in HN1 KD cells. HN1 depletion decreased both 4EBP1 and its S65 phosphorylations together with clear changes in p‐GSK3β^(S9)^. The cells that are treated with LY294002 but not wortmannin exhibited lower expression of 4EBP1 with clearly suppressed S65 phosphorylations. The use of both reagents together resulted in a significant (more than 5‐fold) decrease in 4EBP1 expression consistent with reduced p‐GSK3β^(S9)^. (F) 4EBP1 and its S65 phosphorylation together with clear changes in p‐GSK3β^(S9)^ and p‐GSK3β^(T216)^ phosphorylations were studied w/wo GSK3Bi (SB216763) and proteosome inhibitor treatments for 24 h in HN1 KDcells. HN1 depletion decreased both 4EBP1 and its S65 phosphorylations together with p‐GSK3β^(S9)^ and p‐GSK3β^(T216)^ phosphorylations. The cells that are treated with SB216763 exhibited decreased S65 phosphorylation and expression of 4EBP1. The use of proteosome inhibitors resulted in marginal stabilisation in 4EBP1 level consistent with increased p‐GSK3β^(S9)^. (G) Using immunofluorescent microscopy HN1 colocalization with p‐4EBP1^(S65)^ was examined when HN1 was depleted and overexpressed in comparison to controls in PC3 cells. The speckled distribution of HN1 and p‐4EBP1^(S65)^ phosphorylations colocalized but the distribution did not significantly change.

When HN1 is overexpressed and depleted, the cells are arrested at the S and G2 phases of the cell cycle, respectively. This observation led us to study the global protein synthesis, as we proposed that the histone synthesis might be influenced by HN1 expression. Simply, the lysates from HN1 depleted and HN1 overexpressed asynchronous, or thymidine synchronised cells (T0 and T4) were stained using Coomassie and observed that histone expression is decreased at all time points in HN1 depleted cells (Figure 2A,B). To validate this interesting observation, we further performed treatments with and without Akti (wortmannin) and PI3Ki (LY294002) for 24 h in HN1 OEas well as KD cells, and examined the p‐GSK3β^(S9)^ and p‐4EBP1^(S65)^ phosphorylations. Intriguingly, HN1 OE stabilised both p‐GSK3β^(S9)^ and p‐4EBP1^(S65)^ phosphorylations, independent of inhibitors except for LY294002. In accordance with our previous report that HN1 is associated with p‐GSK3β^(S9)^ [17], the data obtained here supported the notion that HN1 functions in regulating protein synthesis (Figure 2C). Then, p‐4EBP1/2/3 phosphorylations were examined in the presence and absence of GSK3βi and we observed that HN1 OE alone increased the p‐4EBP1/2/3 phosphorylations, suggesting that inhibitory phosphorylation of 4EBP1 is maintained via HN1 expression in addition to GSK3β inhibition. The addition of proteosome inhibitors MG132 and Bortezomib led to the accumulation of HN1 protein in HN1 OE PC3 cells, where the smaller forms of 4EBP1/2/3 phosphorylations decreased while larger forms also increased in comparison to vector controls (Figure 2D). The data suggested that the HN1 effect is more evident than the GSK3β inhibition. The p‐GSK3β^(S9)^ and p‐4EBP1^(S65)^ phosphorylations were also studied w/wo Akti (wortmannin) and PI3Ki (LY294002) treatments for 24 h in HN1 knockdown (KD) cells. HN1 depletion decreased both 4EBP1 expression as well as its S65 phosphorylation where a clear change in p‐GSK3β^(S9)^ was observed. The cells that are treated with LY294002 but not wortmannin also exhibited decreased expression of 4EBP1 with a clear suppression of (S65) phosphorylations. The ucse of both reagents together resulted in a significant (more than 5‐fold) decrease in 4EBP1 expression consistent with reduced p‐GSK3β^(S9)^ (Figure 2E). However, when 4EBP1 as well as p‐4EBP1^(S65)^ together with p‐GSK3β^(S9)^ and p‐GSK3β^(T216)^ phosphorylations were studied w/wo inhibitors, HN1 depletion resulted in a significant decrease in both 4EBP1 and p‐4EBP1^(S65)^ phosphorylation together with GSK3β phosphorylations. Moreover, the cells that are treated with GSK3βi showed the stabilised expression of 4EBP1, whereas p‐4EBP1^(S65)^ phosphorylation marginally decreased consistently with decreased p‐GSK3β^(S9)^ (Figure 2F). However, HN1 depletion decreased the 4EBP1 and S65 phosphorylations almost completely, with a small restoration in MG132 and Bortezomib treatments (Figure 2F). When we examined the subcellular localization of the p‐4EBP1^(S65)^ and HN1 in PC3 cells, we observed that the speckled distribution of the HN1 and 4EBP1^(S65)^ phosphorylations partially colocalized, but the distribution did not significantly change (Figure 2G) in both HN1 depleted and overexpressed cells in comparison to controls. The 4EBP1 speckled spots increased when HN1 was overexpressed. Since 4EBP1 is a well‐known translational regulator and eIF4E activity requires its phosphorylation, the data suggests that the HN1 expression regulates the translation mechanism (apparent from histone levels as control), and its depletion disrupts eIF4E activity for protein synthesis.

### 3.3 HN1 Functions in Regulating Integrity of mTOR‐P70S6K1‐RPS6 Axis

**FIGURE 3:**
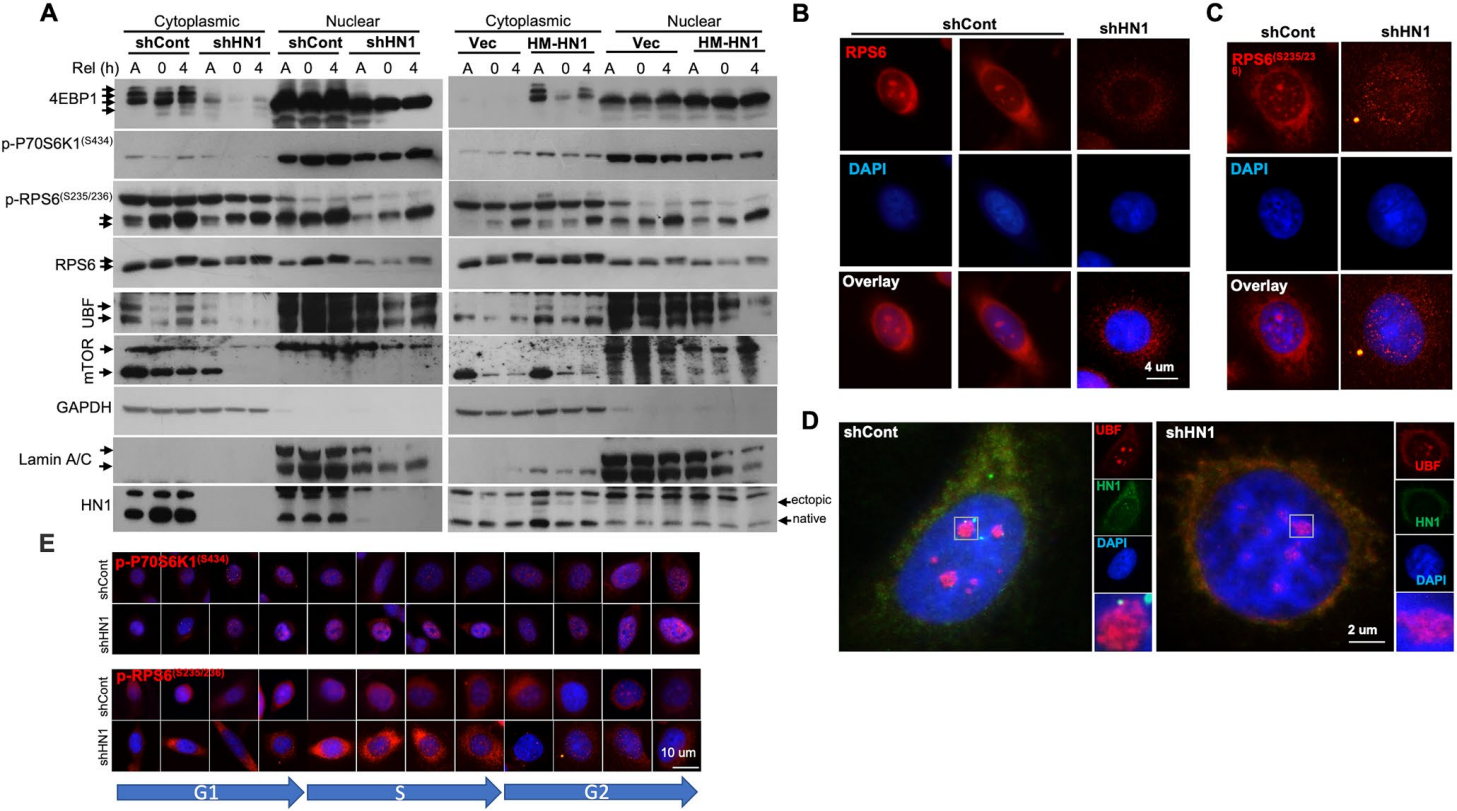
(A) 4EBP1, RPS6, UBF, and mTOR expressions, as well as p‐P70S6K1^(S434)^ and p‐RPS6^(S235/236)^ phosphorylations, were examined in cytoplasmic/nuclear‐fractionated cell lysates in asynchronized and thymidine synchronised HN1 depleted (KD) and HN1 OE PC3 cells. Here, 4EBP1, RPS6, UBF and mTOR expressions as well as p‐RPS6^(S235/236)^ and p‐P70S6K1^(S434)^ phosphorylations significantly decreased in HN1 depleted cells, consistent with increases in HN1 OE cells in comparison to controls. Here A; represents asynchronized cells. Zero represents thymidine synchronised but not released, and four represents 4 h released cells from synchronizations. (B) RPS6, and (C) p‐RPS6^(S235/236)^ were examined in HN1 depleted PC3 cells using immunofluorescent microscopy. (D) When UBF and HN1 colocalizations were examined in both HN1 depleted (KD) and shcontrol PC3 cells, it was found that the nucleolar (proximal to NOR region) localization of UBF expression changed in KD cells in comparison to controls, correlated with western blots. (E) Specked localizations of p‐P70S6K1^(S434)^ and p‐RPS6^(S235/236)^ (same as in C) phosphorylations increased in the S phase clearly indicating a cytoplasmic granulation and retention of p‐RPS6^(S235/236)^ in HN1 KD cells.

A recent report demonstrated that the mTOR pathway (involved directly in protein synthesis) is a target for HN1 activity [15] and 4EBP1 abundance rather than the S65 phosphorylation, is correlated with HN1 expression, so we further proposed that the localization of mTOR components might also be influenced when HN1 is depleted. To uncover the link between HN1 and downstream targets of the mTOR pathway, we studied the 4EBP1, RPS6, UBF and mTOR expressions as well as p‐p70S6K1^(S434)^ and p‐RPS6^(S235/236)^ phosphorylations in subcellular (cytoplasmic/nuclear) fractionated cell lysates in asynchronized and thymidine synchronised HN1 depleted (KD) and HN1 OEPC3 cells. We found that 4EBP1, RPS6, UBF and mTOR expressions as well as p‐RPS6^(S235/236)^ and p‐P70S6K1^(S434)^ phosphorylations significantly decreased in HN1 depleted cells without any treatment, consistent with steep increases in HN1 OE cells in comparison to controls (Figure 3A). When RPS6 and p‐RPS6^(S235/236)^ localizations were examined in HN1‐depleted PC3 cells, a clear speckled distribution was observed around the perinuclear region, using immunofluorescent microscopy (Figure 3B,C, respectively). UBF and HN1 colocalizations were also examined in HN1 KD cells and we found that the nucleolar localization of UBF was marginally distorted in KD cells in comparison to controls (Figure 3D), which correlated with Western blots (Figure 3A). Speckled localization of p‐P70S6K1^(S434)^ and p‐RPS6^(S235/236)^ phosphorylations increased in late S phase of HN1 KD cells (Figure 3E), which was also correlated with western blots from the T4 time point but not with asynchronized cells (Figure 3A). Thus, HN1 contributes to the regulation of protein synthesis through the mTOR‐RPS6 axis, and its depletion dramatically disrupts histone synthesis (Figure 2A,B), hence the nucleosome formation putatively more in proliferating cells at the late S phase, which is evident from altered nuclear size upon HN1 alteration (Figure 1E).

To further study the protein synthesis mechanism upon HN1 alteration directly, p‐P70S6K1^(S434)^ phosphorylation was examined in HN1‐depleted normal mammary epithelial cell line MCF10A. We found that the cells exhibited decreased nuclear localization of p‐P70S6K1^(S434)^ phosphorylation in comparison to controls (Figure S3A). Moreover, when HN1 colocalizations with RPS6, and p‐RPS6^(S235/236)^ and UBF were also examined in HN1‐depleted MCF10A cells, we found that the nucleolar structural integrity was disrupted, which was evidenced by loss of RPS6 localization as well as p‐RPS6^(S235/236)^ phosphorylations and UBF localization at nucleoli in comparison to controls (Figure S3B). We further observed that the HN1 KD resulted in large structural variations in nuclear shape as well as aggregations of nucleolin in these regions in PC3 cells (Figure S4), but interestingly, not in MDA‐MB231 cells (data not shown).

### 3.4 HN1 Associates With mTOR, RPS6 and Nucleolin

**FIGURE 4:**
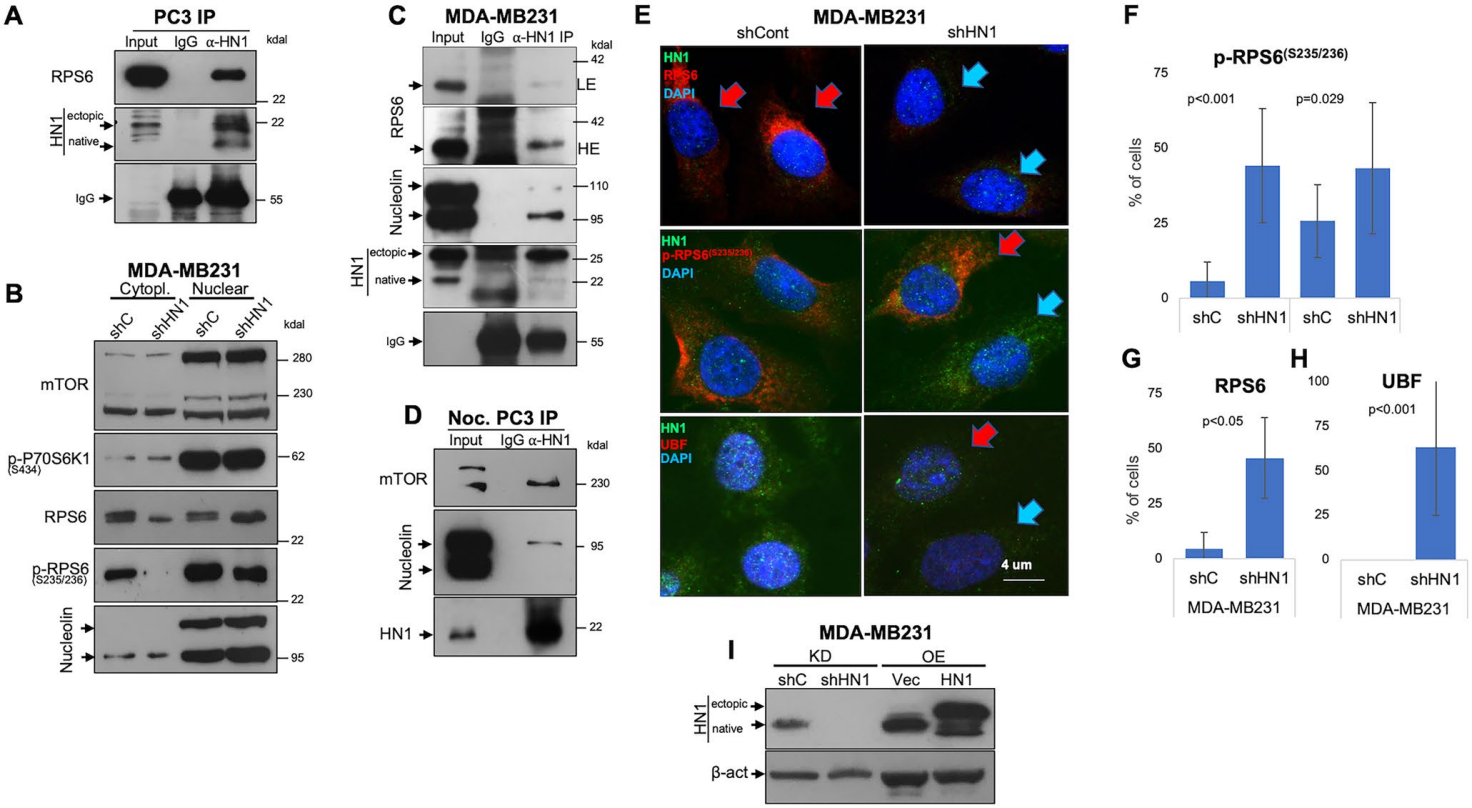
(A) Immunoprecipitations were performed with anti‐HN1 antibody using HN1 overexpressing PC3 cell lysates and, RPS6 interaction was identified by Western blots. (B) Additionally, mTOR and RPS6 expressions as well as p‐RPS6^(S235/236)^ and p‐p70S6K^(S434)^ phosphorylations were examined in cytoplasmic/nuclear‐fractionated cell lysates in asynchronized HN1 depleted (KD) MDA‐MB231 cells. Although there was not a significant variation in mTOR, P70S6K and nucleolin expressions, in control, but not in shHN1 depleted lysates two close bands for RPS6 were observed. Thus, the p‐RPS6^(S235/236)^ phosphorylation was examined and observed that the phosphorylated RPS6 accumulated in nuclear lysates with a clear decrease in HN1 depletion. This implies that nuclear retention occurs due to less phosphorylation in HN1 KD in comparison to controls. (C) Immunoprecipitations were also performed with anti‐HN1 antibody using HN1 overexpressing MDA‐MB231 cell lysates and, RPS6, nucleolin as well as mTOR interactions were confirmed by western blots. All IP experiments were duplicates and immunoblots were at least triplicates. (D) Also, nocodazole (16 h) synchronised PC3 cell lysates were used to enrich the G2 population and the HN1 interactions with mTOR and Nucleolin were demonstrated. (E) HN1 colocalizations with RPS6, and p‐RPS6^(S235/236)^ and UBF were examined in HN1‐depleted MDA‐MB231 cells in comparison to control cells. The cells that were negative for expression were marked with blue and the positives with red arrows. (F) An arbitrary expression cutoff was applied and the negative/positive cells that are expressing the protein of interest were counted from shHN1 cells versus controls. The number of cells counted for p‐RPS6^(S235/236)^ were (*n* = 207) (*p* < 0.001) of MDA‐MB231 and (*n* = 62) (*p* = 0.29) of MCF10A. (G) Again the number of cells counted for RPS6 was *n* = 683 (*p* < 0.05) and (H) it was *n* = 80 for UBF (*p* < 0.001) in MDA‐MB231. The cells were counted using ImageJ software and statistically significant (*p* values/*p* values) differences were found. The scale bar was 4 μm. (I) HN1 depletion and overexpression were confirmed in MDA‐MB231 cells in comparison to controls. B‐actin was also shown as a loading control.

For HN1 involvement in mTOR and protein synthesis pathways, we performed coimmunoprecipitations with anti‐HN1 antibodies using cell lysates from HN1 OE PC3 cells and identified RPS6 interaction using immunoblots (Figure 4A). Additionally, mTOR and RPS6 expressions as well as p‐RPS6^(S235/236)^ and p‐p70S6K^(S434)^ phosphorylations were examined in cytoplasmic and nuclear fractions in HN1‐depleted (KD) MDA‐MB231 cells, and in contrast to PC3 data we found that there was no difference in expressions (Figure 4B). However, in control lysates, two (putative phosphorylation) bands of RPS6 were observed. Consistent with the previous data in Figure 3 in HN1 KD in PC3, there was a single RPS6 form accumulated in nuclear lysates with a clear decrease in (S235/236) phosphorylation. Moreover, nucleolin interactions were found in HN1 OE MDA‐MB231 cell lysates in addition to RPS6 using anti‐HN1 antibody precipitations (Figure 4C). Likewise, HN1 interactions with mTOR and Nucleolin were also demonstrated in nocodazole (16 h) synchronised PC3 cell lysates (Figure 4D), suggesting that the HN1 associations with mTOR components might be cell cycle‐dependent.

The HN1 colocalizations with RPS6, p‐RPS6^(S235/236)^ and UBF were also examined in HN1‐depleted MDA‐MB231 cells in comparison to controls using immunofluorescent microscopy. The cells that are negative for expression were marked with blue and the positives with red arrows. Furthermore, using Image J software, an arbitrary expression cutoff was applied and the negative/positive cells that are expressing the RPS6^(S235/236)^, RPS6 and UBF (depleted/control cells; *n* = 101/106, 383/300, 38/42) were counted from HN1 KD versus controls respectively (Figure 4E). The number of cells was *n* = 40/22 for p‐RPS6^(S235/236)^ in MCF10A cells (Figure 4F). The average expression was calculated as a ratio of negatives/all cells counted, and statistically significant (*p* values/*p* values) differences were found (Figure 4F–H). Since the HN1 expression could be modulated ectopically by experimental settings (Figure 4I), we suggest that the RPS6 expression as well as reduced phosphorylations consequent to its nuclear retention might be due to HN1 depletion, observed in both MDA‐MB231 and PC3 cells in comparison to controls. The association and colocalization studies demonstrated that the HN1 is a component of the mTOR‐P70S6K1‐RPS6 axis, and its depletion results in reduced protein synthesis in these cell lines tested.

### 3.5 HN1 Is Required for Integrity of NOR

**FIGURE 5:**
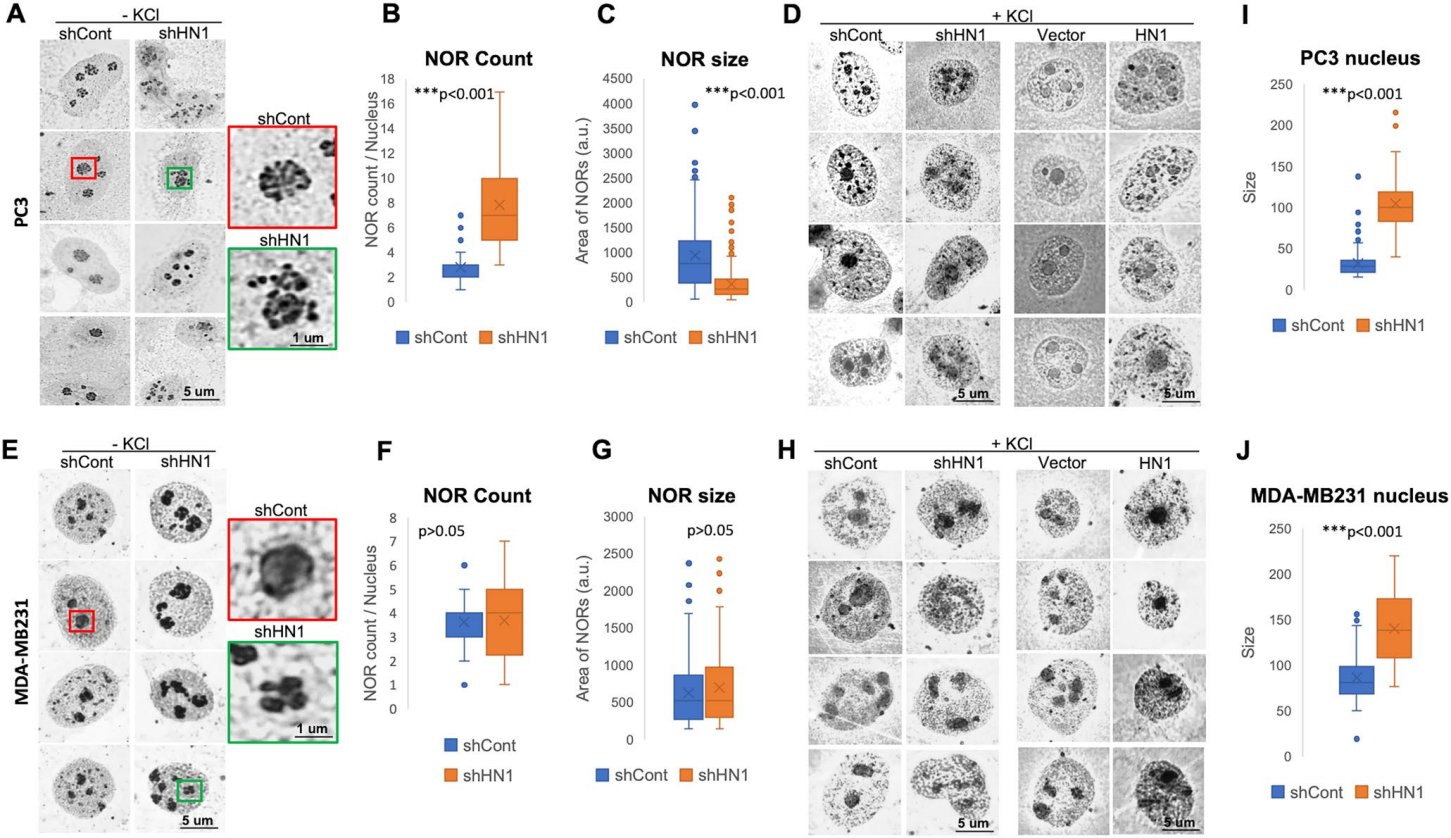
(A–D) NOR staining was performed without KCl treatments in KD cells and with KCl in both conditions, which are the HN1 depletion (KD) as well as HN1 overexpression (OE) in PC3 cells by silver nitrate staining (AgNOR). The staining of NOR regions also demonstrates that the HN1 depletion increases the number of nucleolar structures with severe form changes as well as staining intensity. (B) Quantitation of the NOR count was performed and exhibited a significantly higher number of NOR per nucleolus (*p* < 0.001). (C) Areas of each NOR region were also quantitated and plotted for each nucleus in shHN1 cells versus controls and found that HN1 KD cells exhibited significantly lower mean area for NORs as ratios (*p* < 0.001). (D) HN1 OE cells exhibiting highly granular nucleus having larger nucleoli with denser NOR staining in comparison to controls. (E–H) NOR staining was also performed without KCl treatments in KD cells and with KCl in both conditions of HN1, which are the HN1 depleted (KD) as well as HN1 overexpressed (OE) in the MDA‐MB231 cell line. (F, G) Mean area and the NOR counts were not significantly different (*p* > 0.05). (I) Mean nuclear size was calculated and plotted from PC3 cells, and (J) from MDA‐MB231 cells. HN1 KD cells exhibited significantly larger nuclear sizes in both cell lines (*p* < 0.001). The statistical significance of the data was calculated and given in graphs as either *, ** and *** corresponding *p* > 0.05, 0.01 or 0.001 respectively.

In HN1 KD cells rather than OEs and controls, the cells stained with DAPI showed varied nuclear sizes, as observed in immunofluorescent experiments. Since NOR counts are correlated with translation rate, to investigate the variation in translation rate in these cells, NOR staining was performed using silver nitrate (AgNO3) deposition method with and wo KCl treatments in HN1 depleted (KD) as well as HN1 overexpressed (OE) PC3 (Figure 5A–D) and MDA‐MB231 cells (Figure 5E–H). The results demonstrated that HN1 depletion increased the number of nucleolar structures with severe form changes as well as AgNOR staining intensity in comparison to controls (Figure 5A). HN1 KD PC3 cells exhibited a significantly higher number of NOR per nucleolus (*p* < 0.001) (Figure 5B). When the areas of each NOR region were quantitated for each nucleus, we found that HN1 KD cells exhibited a significantly lower mean area of NORs (*p* < 0.001) (Figure 5C). Despite, HN1 OE PC3 and MDA‐MB231 cells exhibited highly granular nuclei with significantly larger nucleoli (*p* < 0.001) with denser NOR regions, we could not detect a statistically significant difference in NOR ratios in MDA‐MB231 cells (*p* > 0.05), though the AgNOR stained regions were disrupted in nucleolar areas in comparison to controls respectively (Figure 5D,H). Mean nuclear sizes were also calculated and plotted from PC3 cells (Figure 5I) and MDA‐MB231 cells (Figure 5J), demonstrating that the HN1 depletion exhibited significantly larger nuclear sizes in both cell lines (*p* < 0.001). Therefore, we determined that HN1 functions in protein synthesis with evident phenotypes of altered nuclear density and integrity of the nucleolar structure.

### 3.6 HN1 Expression Alters Ribosome Biogenesis

**FIGURE 6:**
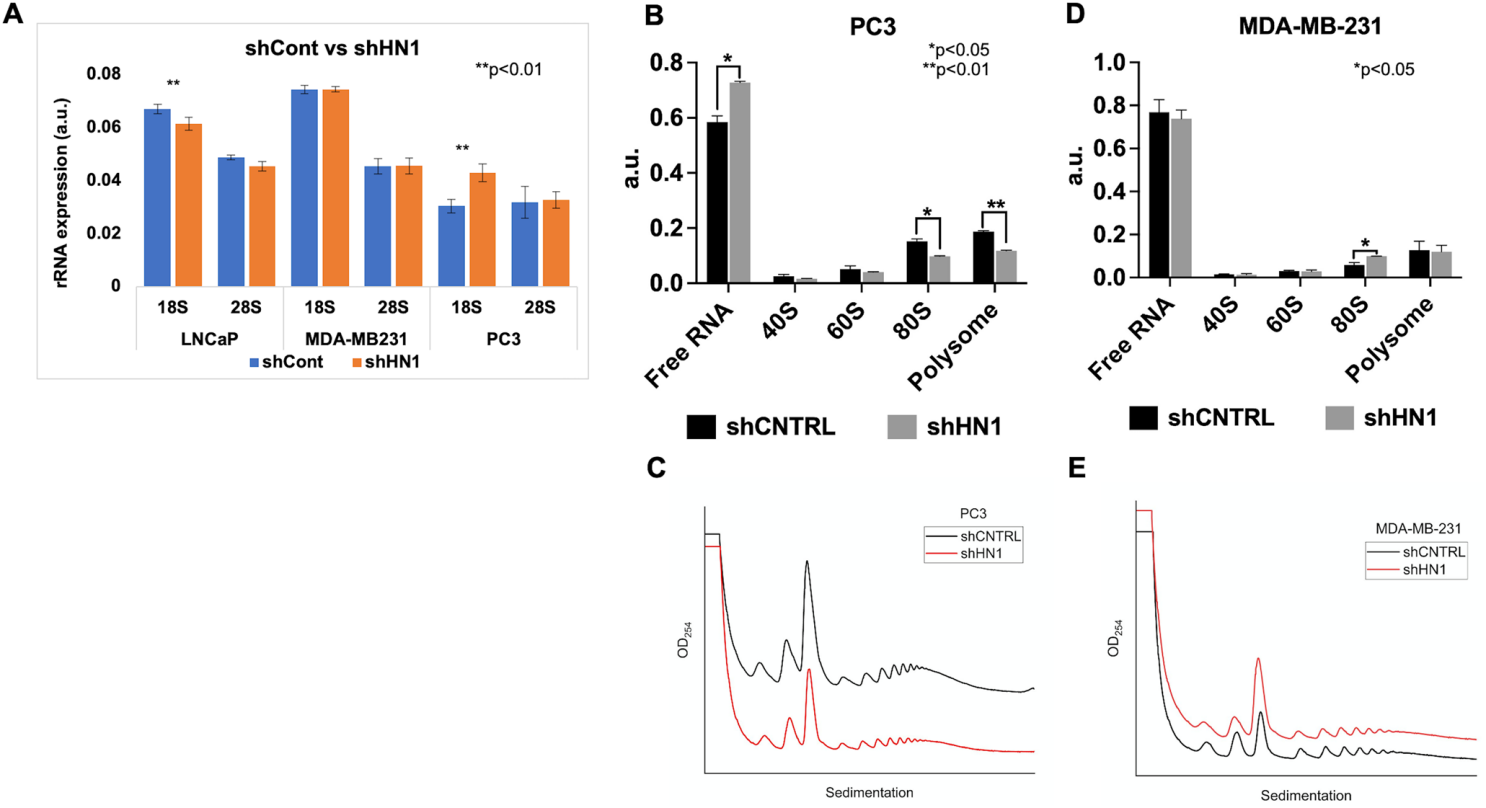
(A) 18S and 28S rRNA levels were relatively quantitated using qRT‐PCR using a double set of specific primers. It was found that the 18S rRNA level slightly but significantly (*p* < 0.001) decreased in LNCaP cells, and increased in PC3 cells, but did not change in MDA‐MB231 cells in HN1 KD samples in comparison to controls. The slight alterations in 28S rRNA level were also observed in both LNCaP and PC3 cell lines but they were not significant. (The data obtained from two different experiments and six to eight replicates for each exp.). (B, C) Same batch of whole cell (2 × 10^6^) lysates were used in polysome analysis and observed that 80S and polysome abundance significantly (*p* < 0.001) decreased in HN1 KD PC3, and (D, E) MDA‐MB231 cells in comparison to controls. (The data were obtained from two different sets of shHN1 experiments and four technical replicates for each cell line). Polysome profiles of control and silenced cells were not overlayed to make it easier to observe subtle differences. The area under each region (e.g., free RNA, 40S, 60S, 80S, and polysomes) was calculated and its percentage in the total area was demonstrated in the graph. The statistical significance of the data was calculated and given in graphs as either *, ** and *** corresponding *p* > 0.05, 0.01 or 0.001 respectively.

To determine if the changes observed in HN1‐altered cells are associated with the maturation of ribosome subunits or rRNA synthesis, we first examined the ribosomal RNA expression. The 18S and 28S rRNA (primer sequences are given in Table 1) expressions were examined in MDA‐MB231, PC3 and LNCaP cells using qRT‐PCR to gain insight into whether the RNA polymerase I activity was influenced by HN1 depletion. We found that the relative quantitation of 18S and 28S ratios in LNCaP as well as in MDA‐MB231 cells was not significantly altered, however, 18S expression was slightly lower in HN1‐depleted LNCaP cells beside 18S rRNA expression was significantly higher in PC3 cells (Figure 6A). In addition, the ratios of small and large subunits of monosome and polysome fractions were examined in CHX‐treated cell lysates from PC3 and MDA‐MB231 cells using ultracentrifugal fractionation. Interestingly, we found that the amount of all ribosome subunits decreased in HN1‐depleted PC3 cells with increased free RNA in comparison to controls (Figure 6B,C). As it was revealed in polysome analysis, it was not the case for HN1‐depleted MDA‐MB231 cells in comparison to controls (Figure 6D,E). The abundance of subunits, monosomes and polysomes inversely correlated with rRNA expression level suggesting that the protein translation rate to NOR integrity is disrupted due to HN1 depletion. Thus, the increased rRNA expression cannot compensate for the polysome formation in cells when HN1 is depleted, subsequently resulting in decreased protein synthesis (Figure 6C–E).

Taken together, the data obtained from rRNA and polysome studies suggest that either HN1's function or itself is required for the regulation of ribosome biogenesis as well as global protein synthesis.

### 3.7 Discussion

The nucleolus has crucial roles in cellular function and structure as it is the designated site for ribosome biogenesis, further regulating replication, protein synthesis and mitotic division [19, 20]. It hosts pre‐rRNA transcription at the FC‐DFC interface, processes it in the DFC, and assembles pre‐ribosomal subunits in the GC [21, 22, 23, 24]. Its size correlates with rRNA abundance, increasing in proliferating cells and many cancers, while reduced rRNA transcription decreases its size [25]. Accordingly, tumour cells generally show an increased size and/or number of nucleoli that can be used as a biomarker for cancer diagnosis [26, 27]. In humans, NORs on acrocentric chromosomes house variable rRNA gene repeats, with transcriptionally active NORs marked by silver staining and Polymerase I‐binding, while silent NORs lack these markers and rely on additional factors for activity [28, 29, 30]. mTOR promotes rRNA synthesis by activating RNA polymerase I via S6K1 and 4E‐BP1, influencing nucleolar size and organisation [31].

HN1 is a highly conserved protein expressed in various tissues [7], including those of the haematopoietic and nervous systems [32]. It has been implicated in diverse cellular processes, such as cell proliferation, differentiation and survival, and is associated with key signalling pathways like Akt and MAPK [1]. HN1 is also linked to tumour progression and metastasis in certain cancers, suggesting its potential role in oncogenesis [8, 9, 33]. Recently, HN1 was shown as a regulator of mTOR signalling in ovarian cancer cells [15]. We previously observed HN1 protein localization in the nucleus where its staining resembled nucleolar structures, prompting us to investigate HN1 in the context of nucleolar and protein synthesis processes.

Here, we investigated the expression and phosphorylation of the mTOR‐RPS6 axis and found that HN1 colocalizes and biochemically associates with mTOR, RPS6 and nucleolin, which are the important components of mRNA translation machinery in eukaryotic cells [34]. As we propose that HN1 might have an important role in ribosome biogenesis, its putative regulatory function could be regulating the abundance of ribosome components, or the protein synthesis as examined using prostate and mammary cancer cell lines. First, we examined the NOR, in terms of expression and phosphorylations as well as HN1 associations of nucleolar components, P70S6K, nucleolin, RPS6 and 4EBP1 in different cell lines. Second, the structural variation of the NORs was quantitated in HN1‐depleted PC3 and MDA‐MB231 cells and observed that the NOR counts were altered significantly in HN1‐depleted cells in comparison to controls. The NOR area changed in PC3 cells but not in MDA‐MB231. Third, the decrease in expression of the ribosome and protein synthesis components shown by western blots of subcellular fractionated lysates correlated with the loss of integrity of the nucleolus with HN1 KDFinally, we examined the expression of 18S and 28S rRNA and found that 18S rRNA expression was slightly higher in PC3 cells when HN1 was depleted. Also, we found that monosome and polysome abundance decreased consistently with increasing free RNA, as revealed in polysome analysis when HN1 was depleted in comparison to controls. Furthermore, HN1 depletion results in dramatic shifts in BrdU incorporation (Figure S5) indicating replication or cell division defects, which may be secondary consequences of altered nuclear size, NORs, protein synthesis mechanism or ribosome biogenesis. Recently, HN1 function in cell division was illustrated as a component of centrosome where it regulates Eg5‐PLK1‐Aurora A axis for maintaining cell division [35], however, evident phenotypes of cell division errors and how HN1's role in protein synthesis mechanism relates to cell division remains unclear. In summation, we revealed that nucleolar structures are distributed unevenly and lose their required associations and decrease the global protein synthesis efficiency when HN1 is depleted. UBF is one of these nucleolar proteins which binds to the polymerase I promoter, and its downregulation results in the interruption of the rRNA synthesis, which is not the case in HN1 depletion. Conversely, we propose that the components of ribosome biogenesis, especially RPS6, Nucleolin and UBF localizations to nucleolar regions are disrupted in the eukaryotic cell when HN1 is depleted. Thus, the data signify that the nucleolar factors require intact HN1 for association and function, which in turn are essential for ribosome biogenesis and function. Taken together, the data suggest that the HN1 expression contributes to ribosome biogenesis whereby its interaction with protein biosynthesis machinery components is crucial for this process. Since HN1 expression is high in solid tumours, anticancer studies might benefit from its depletion or functional loss. Therefore, it is rational to propose that the integrity of the nucleolar structure might be targeted through HN1 depletion as a unique therapeutic approach.

### 3.8 Conclusions

This study highlights the crucial role of HN1 in maintaining nucleolar structure and function, which is essential for ribosome biogenesis and protein synthesis. Depletion of HN1 disrupts the localization and interaction of key nucleolar proteins, such as RPS6, nucleolin and UBF, leading to impaired ribosome production and reduced protein synthesis efficiency. These findings underscore the importance of HN1 in regulating nucleolar integrity and suggest that targeting HN1, particularly in solid tumours where its expression is elevated, could offer a novel therapeutic approach to disrupt ribosome biogenesis and inhibit tumour progression.

## Author Contributions

All the authors contributed to the study conception and design, have agreed to authorship, read and approved the manuscript and have given consent for submission and subsequent publication of the manuscript. Material preparation, data collection and analysis were performed by [Özduman G.], [Javed A.] and [Akçaöz Alasar A.]. The first draft of the manuscript was written by [Korkmaz K.S.] and [Akgül B.] commented on previous versions of the manuscript. It was finalised by [Javed A. and Korkmaz K.S.]. All authors read and approved the final manuscript.

## Ethics Statement

The study does not involve any animal or human subject, or material rather than the authenticated cell lines.

## Conflicts of Interest

The authors declare no conflicts of interest.

## Supporting information


**Figure S1.** (A) Proteosomal degradation of HN1 is blocked when MG132 is used for a short period of time, and CHX treatment decreases HN1 expression marginally, however, larger HN1 forms (putative phosphorylation bands) in both prostate cancer cell lines, PC3 and LNCaP remain higher until 24 h. While MG132 protects HN1 from proteosomal degradation, CHX blocks native HN1’s synthesis, the stability of expression reaches to maxima about 6 h. (B) GSK3B inhibitor SB216763 interfered HN1 stabilisation of both native and ectopic forms with and without double serine phosphorylations at 87–92. B‐actin was used for loading control. (C) The significantly changing cell cycle phase ratios in KD cells in comparison to controls were suppressed by SB216763 treatments in PC3 cells.

**Figure S2.** (A) HN1 depleted PC3 cells exhibit lower growth rate than shControls. (B) Colony formation rate was also found lower in these cells. The assay was conducted in triplicates. (C) Number of colonies counted and plotted as histograms for statistical significancy, where the *t*‐Student’s *t* test was applied (*p* < 0.05). (D) When HN1 is overexpressed (OE) and (E) knocked down by shRNA (KD), cells exhibited lower invasion rate than controls, was measured using real‐time Boyden chambers. Data were collected from tetraplicates (*p* < 0.001 and 3.4 × 10^−7^ respectively). (F) Also, the cellular migration rate was measured and found that it is slower in shHN1 cells than control cells. Scratch assay was conducted in triplicates. (G) The distances that the cells moved were measured and plotted as histograms for statistical significancy, where the student’s *t* test was applied (*p* < 0.05).

**Figure S3.** As a confirmation in normal epithelial cells, (A) first, p‐P70S6K1^(S434)^ phosphorylations were examined in MCF10A cells and observed that nuclear localised (S434) phosphorylations decreased in HN1 depletion in comparison to control cells. (B) Second, HN1 colocalizations with RPS6, and p‐RPS6^(S235/236)^ and UBF were examined using indirect immunofluorescence microscopy. Yellow squares are the regions enlarged at right side of each image and is selected to show the localization and the protein abundance variation in MCF10A cells. The images were captured and analysed using NPlanfluo 100× oil objective (aperture 1.25). DAPI channel shows the nuclear staining. Scale bars are given.

**Figure S4.** Subcellular localization of nucleolin was studied when HN1 was depleted in comparison to controls in PC3 cells using indirect immunofluorescence microscopy and found that the nucleolar localization of the nucleolin disrupted in HN1 KD in comparison to controls. Nucleolin was stained with red (antimouse‐Alexa534 as secondary antibody), and the images were captured and analysed using NPlanfluo 100× oil objective (aperture 1.25). DAPI channel shows the nuclear staining. Scale bars are 6 and 1.2 μm in enlarged images.

**Figure S5.** Cell cycle analysis with BrdU incorporation was performed in HN1 depleted PC3 cells in comparison to controls upon ActD, CHX and TSA treatments for 24 h. Then the cells were stained with anti‐BrdU antibody, RNase treated, PI stained for DNA and flow cytometry analysis performed. It is clearly shown that the HN1 depleted cells are having larger sizes shifted backward with treatments more than control cells.

## Data Availability

The data that support the findings of this study are available from the corresponding author upon reasonable request.
