# Supplementary figures and images for: HN1 Functions in Protein Synthesis Regulation via mTOR‐RPS6 Axis and Maintains Nucleolar Integrity
Source: Cell Prolif. 2025 Jan 13;58(6):e13805. doi: 10.1111/cpr.13805 (PMC12179552; doi:10.1111/cpr.13805)

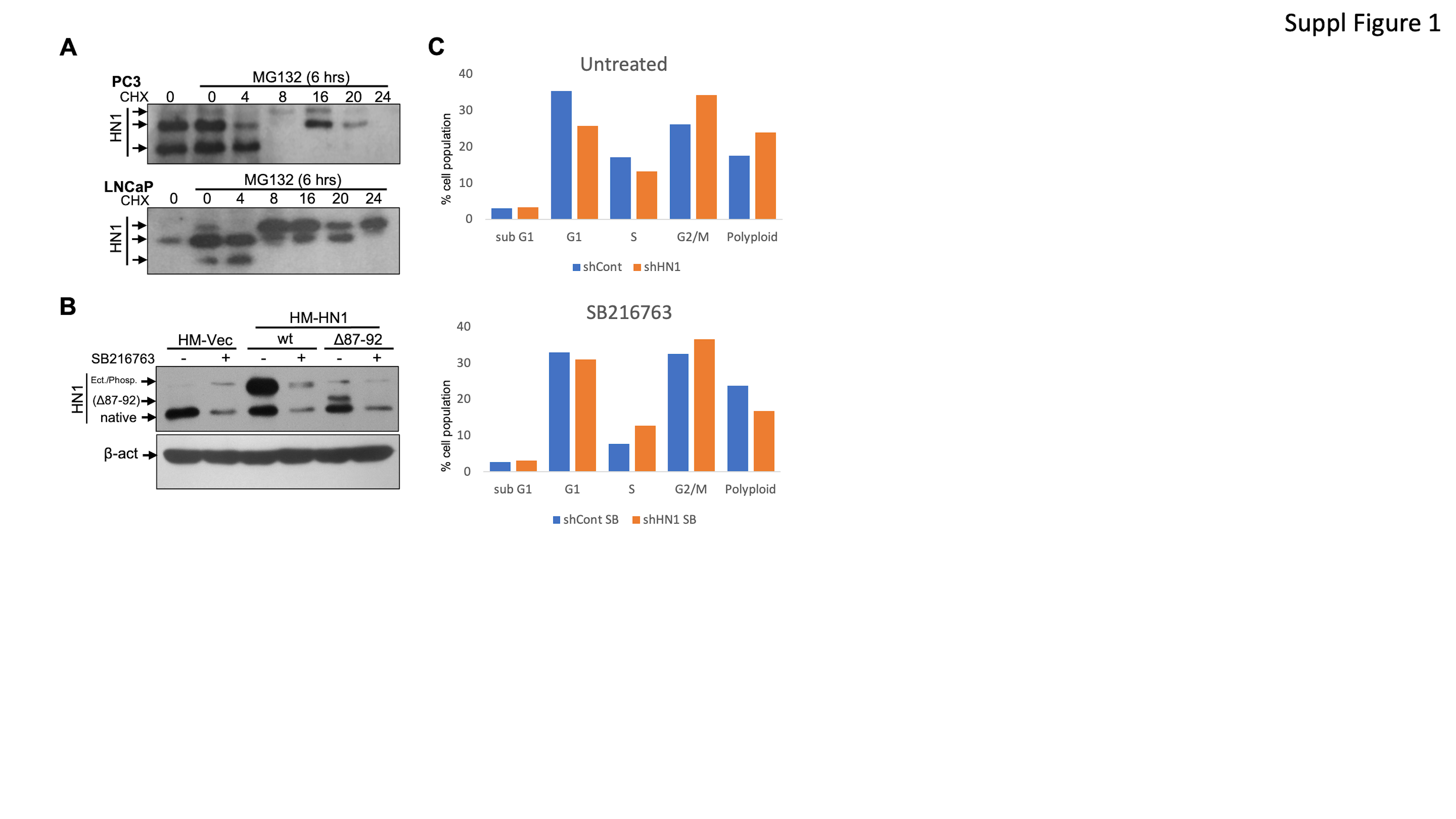

Supplement: Supplementary file 1 — Figure S1. (A) Proteosomal degradation of HN1 is blocked when MG132 is used for a short period of time, and CHX treatment decreases HN1 expression marginally, however, larger HN1 forms (putative phosphorylation bands) in both prostate cancer cell lines, PC3 and LNCaP remain higher until 24 h. While MG132 protects HN1 from proteosomal degradation, CHX blocks native HN1’s synthesis, the stability of expression reaches to maxima about 6 h. (B) GSK3B inhibitor SB216763 interfered HN1 stabilisation of both native and ectopic forms with and without double serine phosphorylations at 87–92. B‐actin was used for loading control. (C) The significantly changing cell cycle phase ratios in KD cells in comparison to controls were suppressed by SB216763 treatments in PC3 cells. [file CPR-58-e13805-s002.tiff]

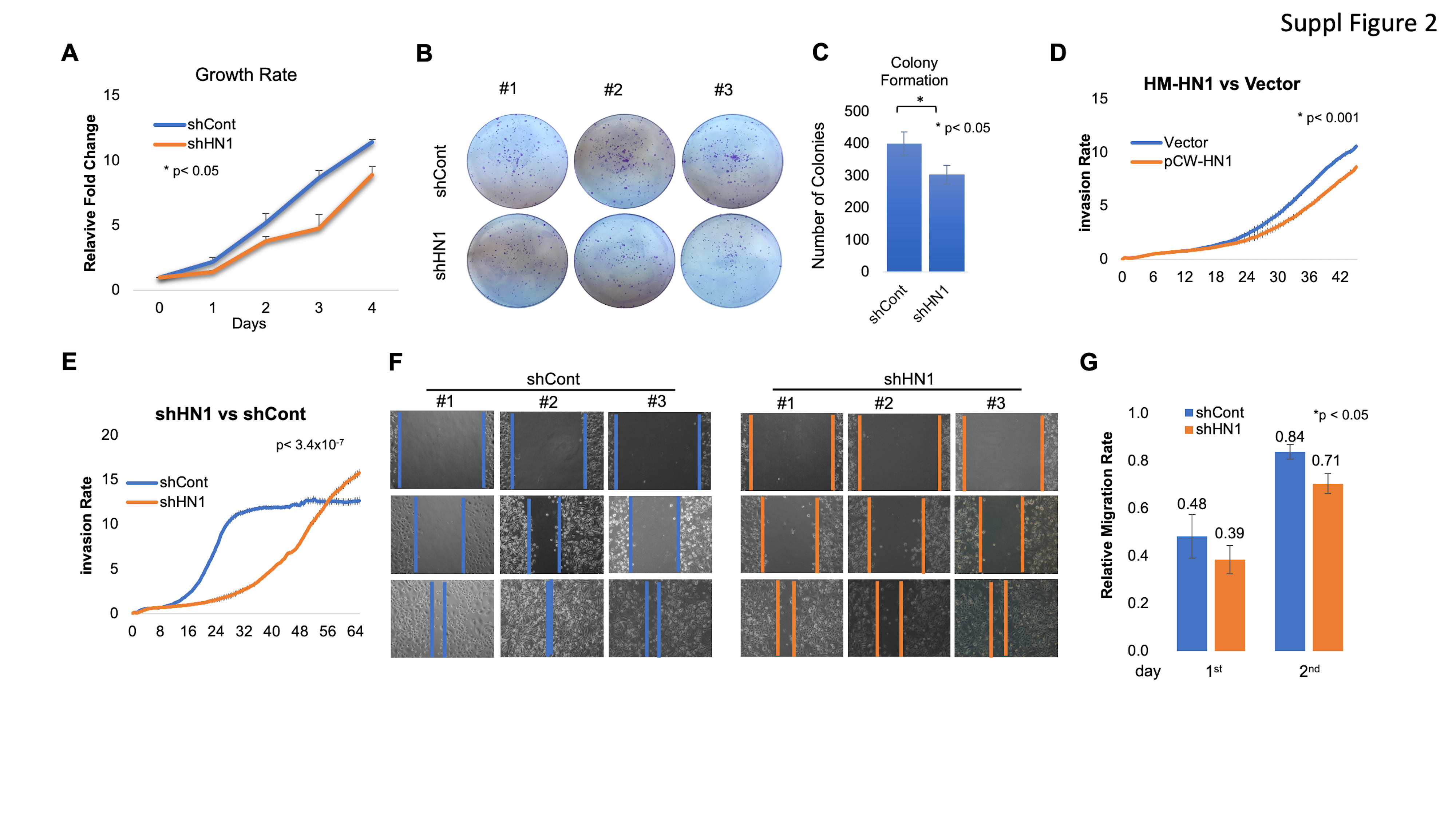

Supplement: Supplementary file 2 — Figure S2. (A) HN1 depleted PC3 cells exhibit lower growth rate than shControls. (B) Colony formation rate was also found lower in these cells. The assay was conducted in triplicates. (C) Number of colonies counted and plotted as histograms for statistical significancy, where the t‐Student’s t test was applied (p < 0.05). (D) When HN1 is overexpressed (OE) and (E) knocked down by shRNA (KD), cells exhibited lower invasion rate than controls, was measured using real‐time Boyden chambers. Data were collected from tetraplicates (p < 0.001 and 3.4 × 10^−7^ respectively). (F) Also, the cellular migration rate was measured and found that it is slower in shHN1 cells than control cells. Scratch assay was conducted in triplicates. (G) The distances that the cells moved were measured and plotted as histograms for statistical significancy, where the student’s t test was applied (p < 0.05). [file CPR-58-e13805-s003.tiff]

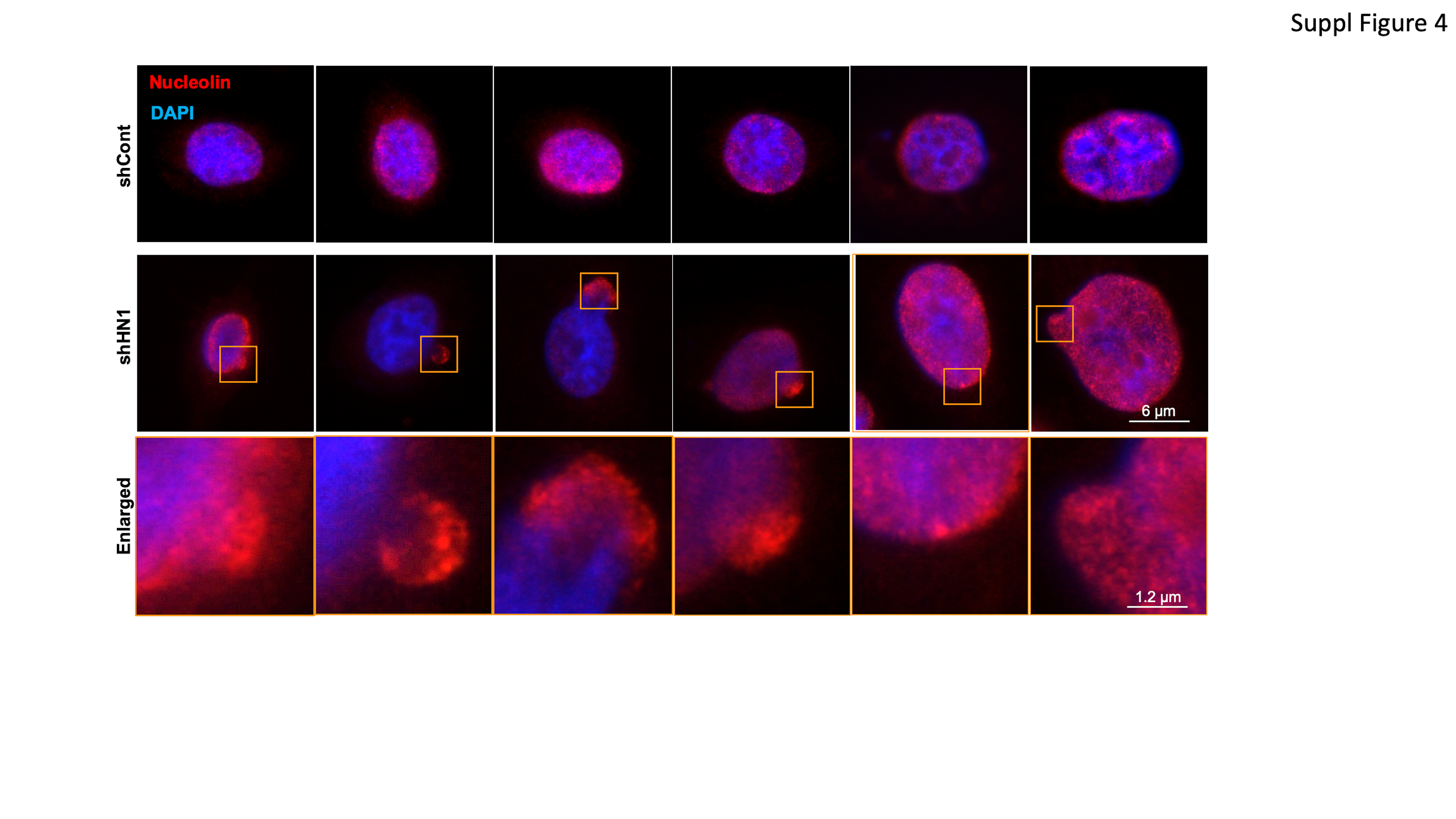

Supplement: Supplementary file 4 — Figure S4. Subcellular localization of nucleolin was studied when HN1 was depleted in comparison to controls in PC3 cells using indirect immunofluorescence microscopy and found that the nucleolar localization of the nucleolin disrupted in HN1 KD in comparison to controls. Nucleolin was stained with red (antimouse‐Alexa534 as secondary antibody), and the images were captured and analysed using NPlanfluo 100× oil objective (aperture 1.25). DAPI channel shows the nuclear staining. Scale bars are 6 and 1.2 μm in enlarged images. [file CPR-58-e13805-s004.tiff]

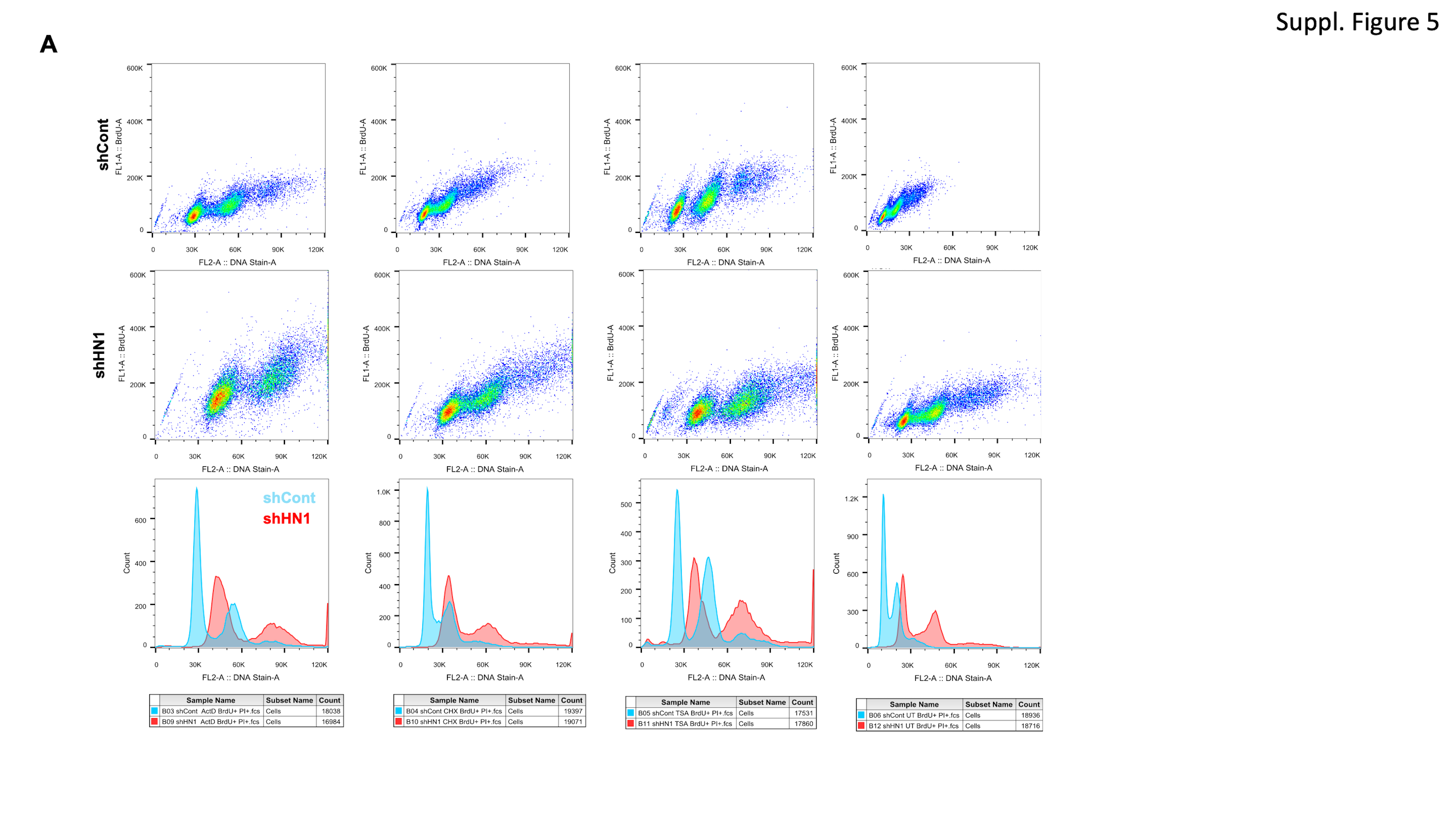

Supplement: Supplementary file 5 — Figure S5. Cell cycle analysis with BrdU incorporation was performed in HN1 depleted PC3 cells in comparison to controls upon ActD, CHX and TSA treatments for 24 h. Then the cells were stained with anti‐BrdU antibody, RNase treated, PI stained for DNA and flow cytometry analysis performed. It is clearly shown that the HN1 depleted cells are having larger sizes shifted backward with treatments more than control cells. [file CPR-58-e13805-s005.tiff]
